# Are Younger Korean Adults Getting Sicker or Getting Treated? Decomposing the Growth in Treated Chronic Disease Prevalence into Disease Burden, Treatment Uptake, and Claims Capture, 2010–2024

**DOI:** 10.3390/healthcare14213729

**Published:** 2026-11-03

**Authors:** Taeuk Kang

**Affiliations:** Department of Biohealth Convergence, College of Natural Sciences, Sungshin Women’s University, 55 Dobong-ro 76ga-gil, Gangbuk-gu, Seoul 01133, Republic of Korea; ethan.kang@sungshin.ac.kr; Tel.: +82-2-920-7585

**Keywords:** chronic disease, treatment uptake, prevalence, decomposition analysis, health surveys, insurance claims, hypertension, diabetes mellitus, hypercholesterolemia, Republic of Korea

## Abstract

**Background/Objectives:** The number of Korean adults treated for chronic diseases has grown fastest at ages 30–49, but whether this reflects more disease or more treatment is unclear. **Methods:** Sex- and age-specific prevalence and treatment rates from the Korea National Health and Nutrition Examination Survey (three-year waves, 2010–2024) were linked to claims-based counts of treated patients with hypertension, diabetes, and hypercholesterolaemia. Changes were decomposed into population, prevalence, treatment rate, and residual claims capture ratio using the logarithmic mean Divisia index with Monte Carlo intervals. **Results:** Among adults aged 30–49 years, treated patients rose by 19% for hypertension, 39% for diabetes, and 123% for hypercholesterolaemia despite a 12% population decline. Survey prevalence was flat for hypertension (13.1% to 13.5%) and diabetes (5.4% to 5.5%), whereas treatment rates rose from 29% to 49% and from 35% to 57%. Treatment uptake accounted for more than the entire net increase for hypertension and diabetes (95% intervals excluding zero), and prevalence for little; for hypercholesterolaemia, prevalence and treatment contributed similarly. At ages ≥ 50, population growth dominated for hypertension and diabetes. Results were robust to alternative specifications, including survey-only decomposition. **Conclusions:** Growth in younger adults under treatment is more consistent with increased detection and treatment uptake than with rising prevalence.

## 1. Introduction

The number of people treated for chronic non-communicable diseases in the Republic of Korea has grown rapidly. Nationwide claims statistics published by the Health Insurance Review and Assessment Service (Healthcare Bigdata Hub; principal-diagnosis-based patient counts) show that, between 2010 and 2025, the number of National Health Insurance (NHI) patients treated for type 2 diabetes doubled and the number treated for dyslipidaemia more than tripled, with the largest relative increases among adults in their thirties and forties. National fact sheets based on the Korea National Health and Nutrition Examination Survey (KNHANES) report that awareness and treatment of hypertension, diabetes, and hypercholesterolaemia have improved markedly over the same period, while noting that management remains poorest among young adults, particularly men [1,2,3,4]. Because people who start treatment for these conditions usually remain on it for decades, growth in the treated population at younger ages may have implications for lifetime treatment exposure and future healthcare expenditure [5,6]. However, expenditure projections that treat demographic ageing as the main driver of chronic disease costs [7] do not identify this component.

A rise in treated prevalence at a given age can arise from three quite different sources. First, it may reflect a true increase in disease occurrence, for example through the rising prevalence of obesity among younger Korean adults and the global increase in young-onset type 2 diabetes [3,8,9]. Second, it may reflect more complete detection and earlier initiation of treatment among people who already have the condition, driven by biennial national health screening, lowered diagnostic thresholds, and expanded treatment indications [10,11,12,13]. Third, it may reflect changes in how care is recorded in claims, such as shifts in principal-diagnosis coding [14]. These sources call for different policy responses. If younger adults are becoming sicker, prevention is the priority; if they are being detected and treated earlier, the key questions are whether the additional treatment is worth its cost and for how long the associated expenditure will accumulate [15]. Claims data alone cannot distinguish between these explanations, because they count only people who present for care.

Population health examination surveys provide the missing denominator. The KNHANES measures blood pressure, fasting glucose, glycated haemoglobin, and blood lipids in a nationally representative sample and records whether respondents have been diagnosed and are taking medication [16]. Serial analyses of KNHANES have documented that hypertension awareness and treatment improved sharply between 1998 and 2007 and then plateaued, with the lowest rates among young adults and men [1,17,18], and that treatment of hypercholesterolaemia has expanded rapidly since 2007 [4]. Global pooled analyses have shown that treatment coverage for hypertension and diabetes has expanded most in high-income countries with universal coverage [19,20], while young adults in the United States continue to show markedly lower awareness and treatment than older adults [21,22]. However, to our knowledge, survey-based and claims-based measures have not been combined to apportion the growth in treated populations between disease burden and treatment uptake, nor have the age groups with the fastest expansion been examined separately.

In this study we linked KNHANES estimates of prevalence and treatment rates to nationwide claims-based counts of treated patients with hypertension, diabetes, and hypercholesterolaemia by sex and age group over five survey waves from 2010 to 2024 (four waves, 2013–2024 for diabetes). We decomposed the change in the number of treated patients into the contributions of population size, survey-measured prevalence, the treatment rate among people with the condition, and a residual capture ratio between claims and survey measures. We operationally defined younger adults as those aged 30–49 years, the youngest age bands for which survey estimates of prevalence and treatment are sufficiently precise. Our aim was to determine, particularly for this group, whether the growth in the number of people under treatment is more consistent with more disease or with more treatment.

## 2. Materials and Methods

### 2.1. Design and Data Sources

This was a repeated cross-sectional ecological study linking two nationwide data sources at the level of sex and 10-year age group. Survey-based measures were taken from the official KNHANES trend tables published by the Korea Disease Control and Prevention Agency with the 2024 statistics (Korea Health Statistics 2024). For each indicator, these tables report the sample size, the weighted estimate, and its standard error by sex and age group, accounting for the complex survey design [16]. Prevalence is reported annually; awareness and treatment rates are reported by three-year survey wave because the number of people with each condition in a single year is too small for stable age-specific estimates. Values were extracted programmatically from the official workbooks and checked against the 2017–2023 editions: all 1950 overlapping estimates of hypertension and diabetes prevalence, awareness, and treatment at ages ≥ 30 years, and their standard errors, were identical.

Claims-based counts of treated patients were obtained from the Healthcare Bigdata Hub of the Health Insurance Review and Assessment Service (HIRA), which reports the annual number of de-duplicated patients with a principal diagnosis of essential hypertension (ICD-10 I10), type 2 diabetes mellitus (E11), and disorders of lipoprotein metabolism (E78) by sex and 5-year age group. We used principal-diagnosis counts because they are the only disease-specific patient counts published by age and sex for the whole period. They exclude patients whose condition is recorded only as a secondary diagnosis; this limitation is absorbed by the capture ratio described below. Mid-year population by sex and age was obtained from Statistics Korea. In these tables, a patient who receives care in two age groups within a calendar year (for example, before and after a birthday that crosses an age-group boundary) is counted in both; consequently, the sum of the age-specific counts exceeded the de-duplicated national total by 10–14% for I10 and E11 and by 4–8% for E78 over the study period; sex-specific totals are not affected. Because this inflation varies little over time, it enters the decomposition almost entirely through the level of the capture ratio; a sensitivity analysis that removes it is described in Section 2.4.

### 2.2. Definitions

All KNHANES definitions follow the official indicators. Hypertension was defined as systolic blood pressure ≥ 140 mmHg, diastolic blood pressure ≥ 90 mmHg, or current use of antihypertensive medication; awareness as having been diagnosed by a physician; and treatment as taking antihypertensive medication on 20 or more days per month. Diabetes was defined as fasting plasma glucose ≥ 126 mg/dL, glycated haemoglobin ≥ 6.5%, physician diagnosis, or use of glucose-lowering agents or insulin; awareness as physician diagnosis; and treatment as current use of glucose-lowering agents or insulin. Hypercholesterolaemia was defined as total cholesterol ≥ 240 mg/dL after fasting for at least 8 h or current use of lipid-lowering medication; awareness and treatment were defined analogously. Awareness and treatment rates are expressed as a percentage of people with the condition. Medication use enters the prevalence definitions of all three conditions, so that treatment and measured prevalence are not fully independent. For hypertension and diabetes, pharmacological treatment is generally initiated in people who meet the diagnostic thresholds [10,11], so the medication criterion mainly retains people who already had the condition and are controlled on treatment. If wider treatment nonetheless raised measured prevalence, part of the treatment effect would be attributed to the prevalence component, making our conclusions for these conditions conservative. For hypercholesterolaemia, lipid-lowering medication is frequently prescribed on the basis of overall cardiovascular risk to people whose total cholesterol is below 240 mg/dL [4], so an increase in lipid-lowering treatment raises measured prevalence directly as well as the treatment rate. The claims category E78 (disorders of lipoprotein metabolism and other lipidaemias) is also broader than the survey definition, which is based on total cholesterol alone: it includes hypertriglyceridaemia, mixed hyperlipidaemia, and low high-density lipoprotein cholesterol. The capture ratio for this condition therefore also absorbs differences in disease scope, and the survey-only decomposition described in Section 2.4 provides a check that is independent of the claims definition. The KNHANES diabetes indicator does not distinguish type 1 from type 2 diabetes, whereas the claims counts refer to type 2 diabetes (E11); because type 1 diabetes accounts for a small fraction of adult diabetes in Korea, this mismatch is unlikely to affect the results materially.

Five survey waves were used: 2010–2012, 2013–2015, 2016–2018, 2019–2021, and 2022–2024, corresponding to the fifth to ninth KNHANES cycles. Because the glycated haemoglobin criterion for diabetes was introduced in 2011 and age-specific awareness and treatment indicators under this definition are available only from the 2013–2015 wave onward, the diabetes analysis covers 2013–2024. Age groups were 30–39, 40–49, 50–59, 60–69, and ≥70 years; adults aged 19–29 years were excluded because the number of survey respondents with each condition was too small. Claims-based patient counts and population were aggregated from 5-year to 10-year age groups by simple summation and averaged over the three calendar years of each wave. All decomposition calculations were performed separately within each sex–age cell, and the contributions were then summed across sexes and age groups; this is valid because log-mean Divisia index(LMDI) contributions are additive in patient counts. Survey-based rates pooled across sexes in Table 1 are weighted by the number of survey respondents (for prevalence) or the number of respondents with the condition (for awareness and treatment) in each cell. Survey prevalence for a wave was calculated as the sample-size-weighted mean of the three annual estimates. Because the published annual estimates are already weighted to the population by the survey design, the choice of weights for combining the three years affects only the relative influence of each year; population-weighted and unweighted means were examined in a sensitivity analysis.

### 2.3. Decomposition Framework

For each disease, sex, and age group, the number of claims-based treated patients in a wave was expressed as an identity of four multiplicative components (Equation (1)):

(1) $$n=N\cdot \frac{n_{s}}{N}\cdot \frac{n_{1}}{n_{s}}\cdot \frac{n}{n_{1}}=N\cdot P\cdot T\cdot C$$

where *n* is the number of treated patients in claims, *N* is the population, *P* is the survey-measured prevalence, *T* is the treatment rate among people with the condition, and *C* is the claims capture ratio, a residual claims-to-survey concordance ratio defined as the claims-based treated prevalence (*n*/*N*) divided by the survey-based treated prevalence (*P* × *T*). *C* absorbs all differences between the two measurement systems, including differences in treatment definitions, principal-diagnosis coding practices, multimorbidity, health-care-seeking, and intermittent treatment, and should not be interpreted as a direct measure of coding completeness. Because *C* is obtained as a residual rather than measured independently, its contribution is interpreted only as the part of the change in claims-based counts that is not accounted for by population and the survey-based components. It can exceed 1, for example where claims count people who visit for the condition without taking medication regularly or count a patient in more than one age group, and fall below 1, for example where treated patients are recorded under a different principal diagnosis. Validation studies in Korea have shown that claims-based definitions of diabetes agree well with KNHANES criteria when prescription codes are combined with diagnosis codes, but that diagnosis codes alone identify a broader population [14].

Changes in treated patients between consecutive waves were decomposed using the additive logarithmic mean Divisia index (LMDI-I) [23,24], chosen because it provides an exact additive decomposition of changes in a multiplicative identity without an unexplained residual and is invariant to the order of factors (Equation (2)):

(2) $$∆n^{X}=L(n_{1},n_{0})\ln\frac{X_{1}}{X_{0}}$$

where *L*(*a*, *b*) = (*a* − *b*)/(*ln a* − *ln b*) is the logarithmic mean and *X* denotes each component. Alternatives include the Laspeyres and Paasche index decompositions, which leave an interaction residual, and the Shapley decomposition, which is also exact but requires averaging over all orderings of the factors in each period [23]. LMDI-I additionally gives contributions that are consistent in aggregation, so that contributions estimated within sex–age cells can be summed across cells and across chain-linked periods [23]; we confirmed that the Shapley method gives practically identical results (Section 3.3). Contributions were chain-linked across waves and summed over sexes and age groups into three age bands (30–49, 50–69, and ≥70 years). Each contribution is reported in thousands of patients and as a percentage of the total change; because positive and negative components offset one another, individual contributions can exceed 100% of the net change.

### 2.4. Uncertainty and Sensitivity Analyses

Because survey prevalence and treatment rates are estimated with sampling error, we propagated their published standard errors by Monte Carlo simulation: in each of 2000 replications, every annual prevalence estimate and every wave-specific treatment rate was drawn from a normal distribution with the published mean and standard error, the capture ratio was recalculated, and the decomposition was repeated. Draws for different years and survey waves are independent because they come from separate survey samples. Within a cell and wave, however, prevalence and treatment rates are estimated from overlapping respondents and their covariance is not published; we therefore assumed independence in the main analysis and examined correlations of +0.5 and −0.5 between the prevalence and treatment draws as a sensitivity analysis. Normal draws can fall outside the unit interval: in 17 of 140 disease–wave–sex–age cells, all aged 30–39 years, at most 2.6% of the draws of an annual prevalence or treatment rate were negative, and the pooled prevalence and the treatment rate were bounded below at 0.0001 and 0.001, respectively. As a sensitivity analysis, we instead drew each proportion on the logit scale, with standard errors transformed by the delta method, which keeps all draws within the unit interval. We report the median and the 2.5th and 97.5th percentiles of each contribution. Population and claims counts are complete enumerations and were treated as fixed. We conducted five further sensitivity analyses. First, we replaced the treatment rate with the awareness rate, so that the third component represents detection and the capture ratio absorbs the step from diagnosis to treatment; we also extended the identity to five components by writing the treatment rate as the product of the awareness rate and the ratio of treatment to awareness, which separates detection from treatment among people already diagnosed. Second, annual prevalence estimates were pooled with population weights or with equal weights instead of survey sample sizes. Third, we decomposed the survey-implied number of treated people (*N* × *P* × *T*) into population, prevalence, and treatment components without using claims data, which tests whether the conclusions depend on the claims definitions or on the capture ratio. Fourth, the decomposition was repeated with the Shapley method. Fifth, claims-based counts were rescaled within each disease, year, and sex so that the age-specific counts summed to the de-duplicated total, assuming that duplicate counting is proportional across age groups. Analyses were performed in Python 3.12 and R 4.3; the analytic dataset and code are provided as Supplementary File S1.

## 3. Results

### 3.1. Trends in Prevalence, Treatment, and Treated Patients

**Figure 1:**
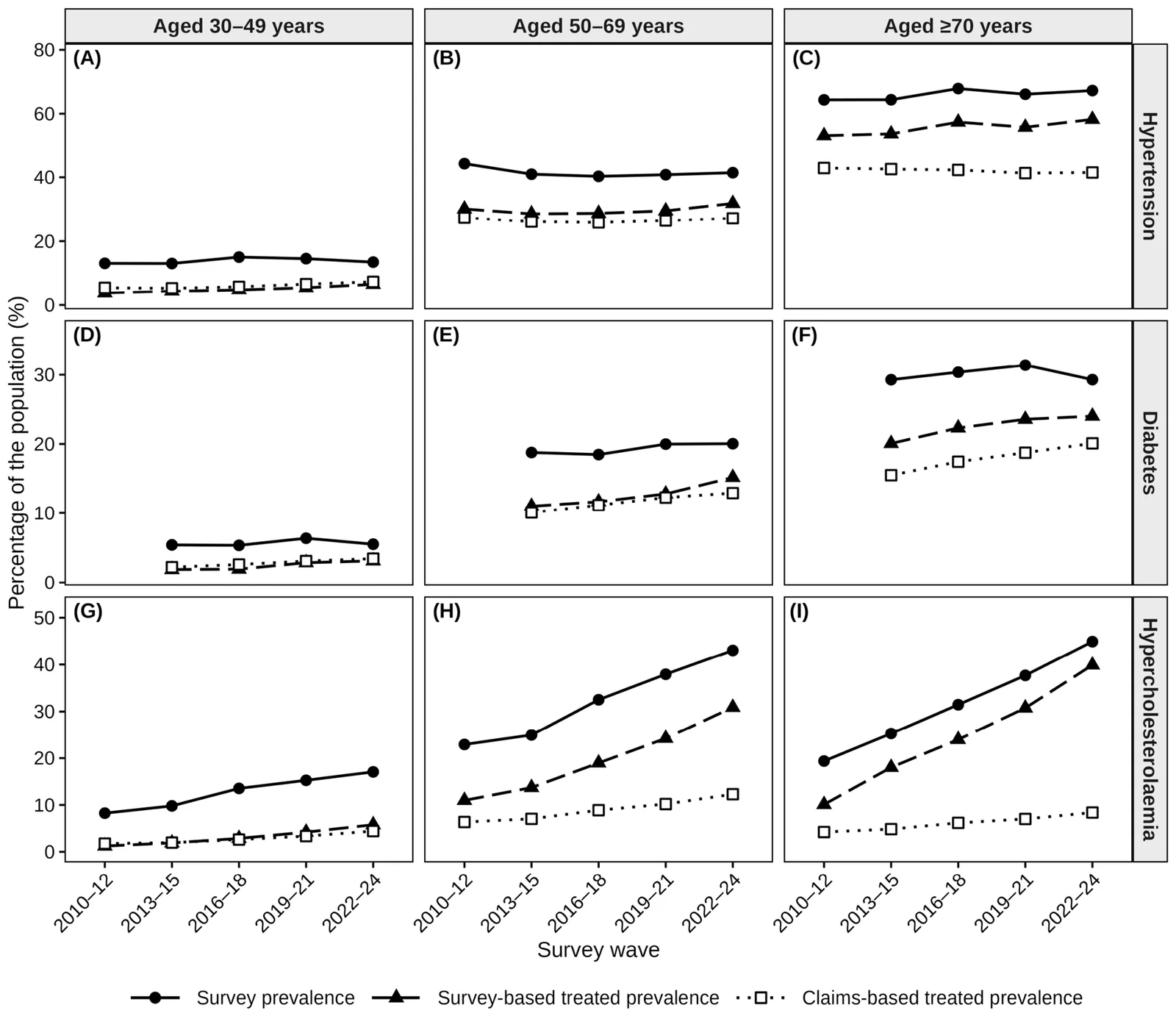
Survey-measured prevalence, survey-based treated prevalence (prevalence × treatment rate), and claims-based treated prevalence by survey wave for hypertension (**A**–**C**), diabetes (**D**–**F**), and hypercholesterolaemia (**G**–**I**) among adults aged 30–49, 50–69, and ≥70 years, Korea, 2010–2024. Source: KNHANES trend tables and HIRA claims statistics.

**Figure 2:**
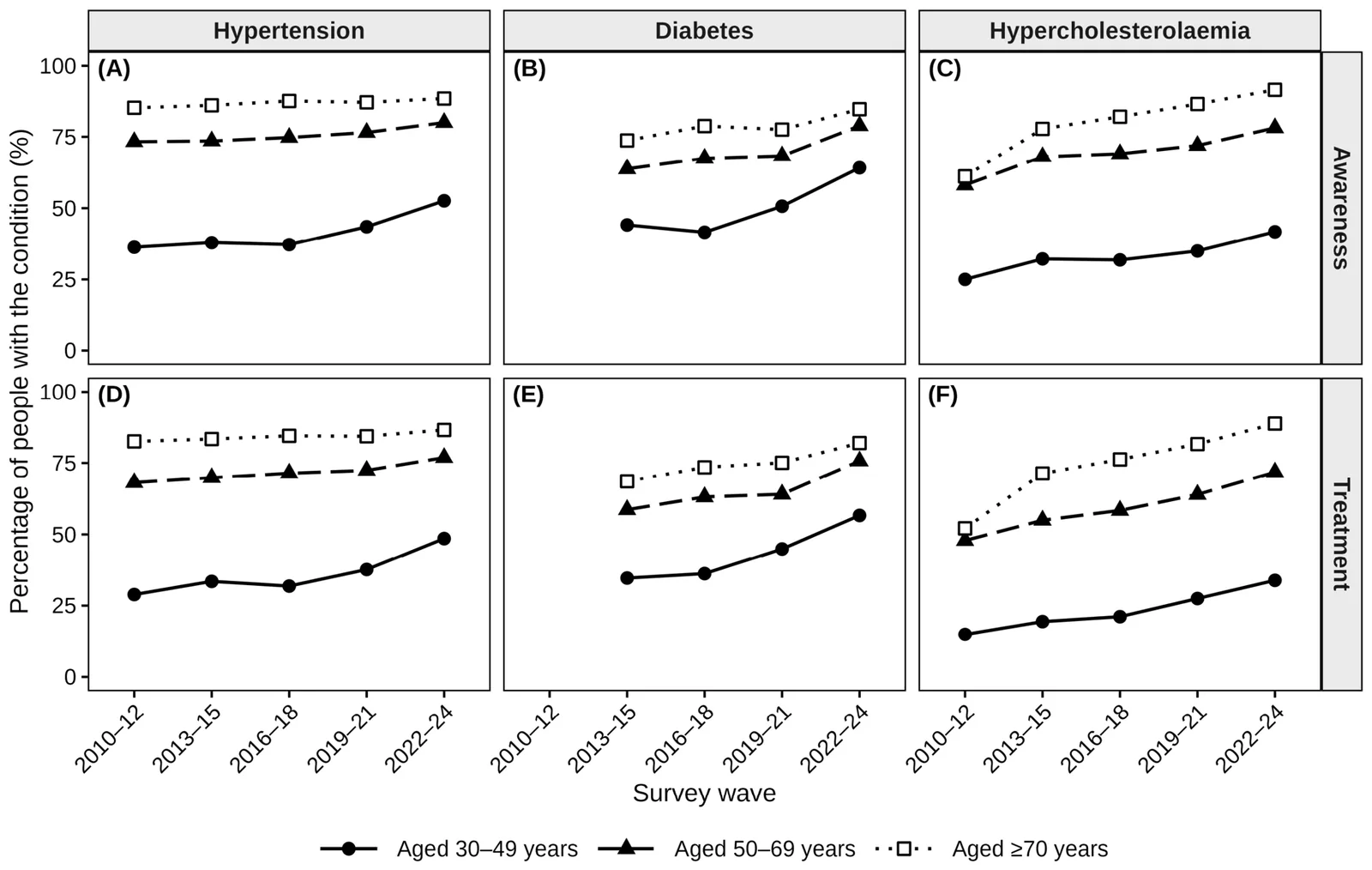
Awareness (**A**–**C**) and treatment (**D**–**F**) rates among people with hypertension, diabetes, and hypercholesterolaemia by survey wave and age group, KNHANES 2010–2024.

Table 1 summarises the components at the first and last waves. Among adults aged 30–49 years, the population fell by 12% between 2010–2012 and 2022–2024, yet the number of patients treated for hypertension rose from 916,000 to 1,089,000 (+19%), for diabetes from 367,000 (2013–2015) to 511,000 (+39%), and for hypercholesterolaemia from 295,000 to 657,000 (+123%). Survey prevalence at these ages was essentially unchanged for hypertension (13.1% to 13.5%) and diabetes (5.4% to 5.5%) and doubled for hypercholesterolaemia (8.3% to 17.1%). For hypercholesterolaemia, however, prevalence and treatment are not independent, because medication use is part of the prevalence definition. In contrast, treatment rates among people with the condition rose steeply: from 28.9% to 48.6% for hypertension, from 34.7% to 56.6% for diabetes, and from 14.9% to 33.9% for hypercholesterolaemia (Figure 1 and Figure 2). Awareness rose in parallel, from 36% to 53%, 44% to 64%, and 25% to 42%, respectively.

At ages 50–69 years, the population grew by 42% and treated patients by 41% for hypertension, 64% for diabetes, and 175% for hypercholesterolaemia. Survey prevalence of hypertension declined slightly (44.3% to 41.4%), that of diabetes was stable (18.8% to 20.0%), and that of hypercholesterolaemia nearly doubled (22.9% to 43.1%); treatment rates rose from 68.1% to 76.9%, 58.6% to 75.7%, and 47.9% to 71.8%, respectively. At ages ≥ 70 years, treatment rates for hypertension were already above 80% throughout, whereas those for hypercholesterolaemia rose from 52.1% to 88.8%.

The claims capture ratio declined in almost every group (Table 1). Among adults aged 30–49 years it fell from 1.44 to 1.12 for hypertension and from 1.43 to 0.77 for hypercholesterolaemia, indicating that survey-based treated prevalence grew faster than the number of patients recorded in claims under the corresponding principal diagnosis. The ratio itself cannot identify the source of this discrepancy. Part of the level of the ratio, but little of its trend, reflects duplicate counting of patients across age groups in the claims tables (Section 2.1).

### 3.2. Decomposition of the Change in Treated Patients

**Figure 3:**
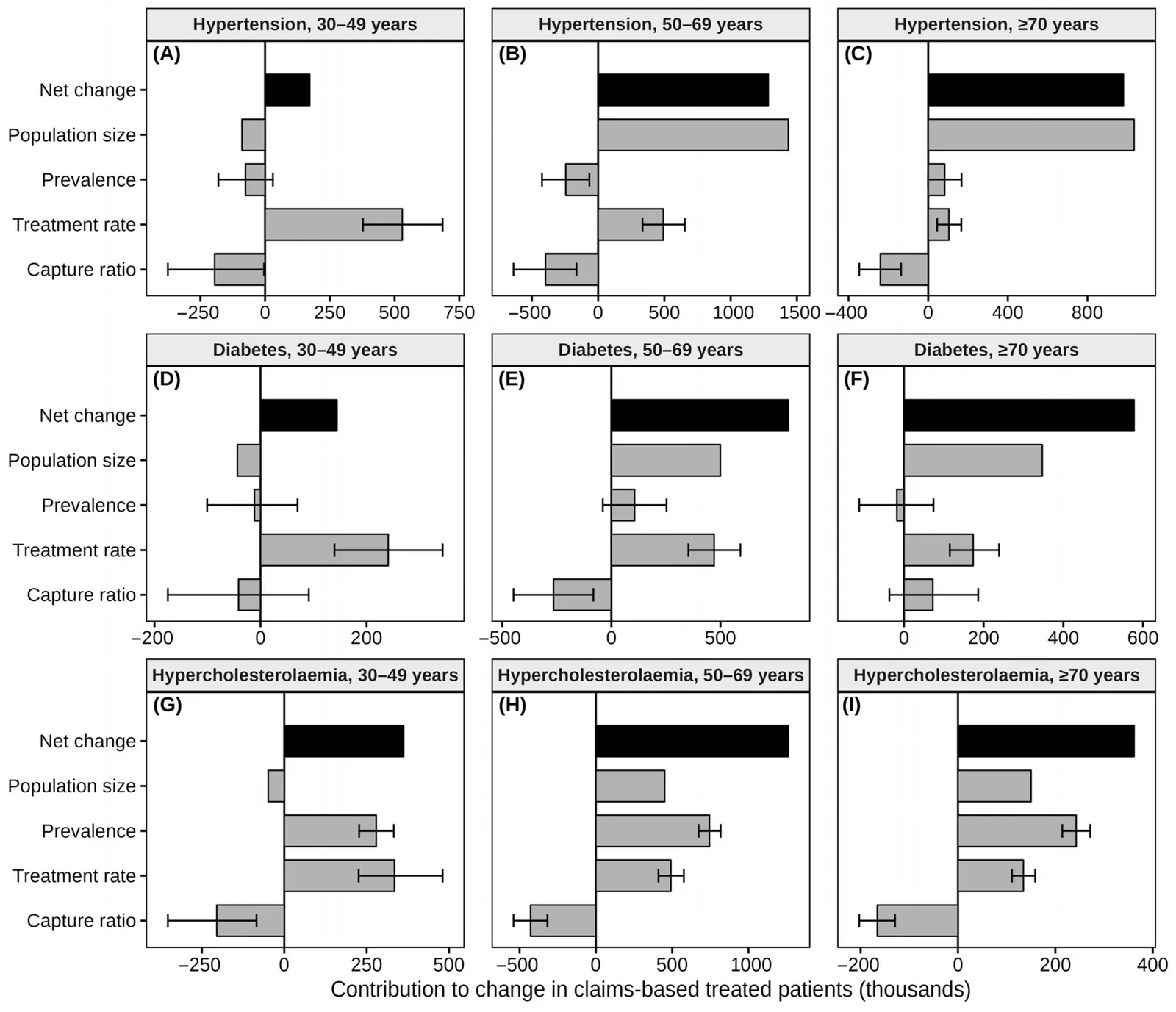
Contributions of population size, survey-measured prevalence, treatment rate, and claims capture ratio to the change in claims-based treated patients between the first and last survey waves, by disease and age band (thousands of patients). In each panel, the black bar shows the net change and the grey bars its four components; error bars are Monte Carlo 95% intervals (population size is treated as fixed). The wide intervals for prevalence and capture among adults aged 30–49 years with diabetes reflect the small number of survey respondents with diabetes at these ages. Hypertension and hypercholesterolaemia: 2010–2012 to 2022–2024; diabetes: 2013–2015 to 2022–2024.

Table 2 and Figure 3 show the decomposition. Among adults aged 30–49 years, the increase of 172,000 patients treated for hypertension was the net result of a negative population contribution (−89,000), a negative and statistically uncertain prevalence contribution (−76,000; 95% interval −180,000 to 30,000), a large positive treatment-rate contribution (+530,000; 378,000 to 686,000), and a negative capture contribution (−195,000; −375,000 to −4000). Diabetes showed the same pattern: of the 144,000 additional treated patients, prevalence contributed −12,000 (−101,000 to 70,000) and treatment uptake +241,000 (140,000 to 344,000). For diabetes at these ages, the intervals for the prevalence and capture components were wide and included zero, reflecting the small number of survey respondents in their thirties with diabetes; the treatment component, however, was positive throughout its interval, so the qualitative conclusion for this group does not depend on the precision of the prevalence estimate. For hypercholesterolaemia, both prevalence (+279,000; 227,000 to 333,000) and treatment uptake (+334,000; 226,000 to 481,000) contributed substantially to the increase of 362,000 patients, offset by a negative capture contribution (−205,000).

At ages 50–69 years, population growth was the largest component for hypertension (112% of the change) and diabetes (62%), with treatment uptake contributing 38% and 58%, respectively, and prevalence contributing negatively for hypertension (−19%) and marginally for diabetes (13%; interval including zero). For hypercholesterolaemia, rising prevalence (59%) and treatment uptake (39%) both exceeded population growth (36%). At ages ≥ 70 years, population growth was the largest component for hypertension (106%) and diabetes (60%); for diabetes, treatment uptake also contributed substantially (30%; 95% interval 115,000 to 239,000), and the capture ratio contributed 13% with an interval including zero. For hypercholesterolaemia at these ages, prevalence (67%) and treatment uptake (37%) together exceeded the contribution of population growth (42%). The capture ratio contributed negatively in every group except diabetes at ages ≥ 70 years.

### 3.3. Sensitivity Analysis

When awareness replaced treatment as the third component, detection accounted for 215% of the net change among adults aged 30–49 years for hypertension, 132% for diabetes, and 55% for hypercholesterolaemia, with prevalence contributing −43%, −8%, and 77%, respectively (Supplementary Table S1). In the five-component extension, the treatment contribution in this age group was split between rising awareness (215%, 132%, and 55%) and a rising ratio of treatment to awareness (90%, 33%, and 35%; Supplementary Table S2); the proportion of aware people who were treated rose from 0.80 to 0.92 for hypertension, from 0.79 to 0.88 for diabetes, and from 0.59 to 0.81 for hypercholesterolaemia. Increases in diagnosis and in treatment among the diagnosed therefore both contributed, with diagnosis accounting for 61–80% of the combined contribution; because both rates are estimated from small numbers of people with each condition, this split should be regarded as approximate.

The results were robust to the other specifications (Supplementary Tables S3 and S5). Pooling annual prevalence with population or equal weights changed the prevalence and capture contributions by at most 4.4 percentage points of the net change and left the treatment contributions unchanged. Logit-scale draws changed the median contributions by at most 4.0 percentage points, and correlations of ±0.5 between prevalence and treatment draws by at most 4.4 points; the correlations mainly affected the width of the capture-ratio intervals, so that with a positive correlation the interval for the capture contribution among adults aged 30–49 years with hypertension included zero (−404,000 to 35,000). In every specification, the 95% interval of the treatment contribution excluded zero in all disease–age groups. The Shapley decomposition differed from LMDI by at most 5.1 percentage points. Rescaling the claims counts for duplicate counting across age groups reduced the counts by 4–12% but changed the percentage contributions by at most 4.1 points.

In the survey-only decomposition, which does not use claims data, the survey-implied number of treated people aged 30–49 years increased by 303,000 for hypertension, 155,000 for diabetes, and 634,000 for hypercholesterolaemia. The treatment-rate component accounted for 147%, 137%, and 60% of these increases (95% intervals 313,000 to 570,000; 121,000 to 295,000; and 275,000 to 485,000), whereas the prevalence component accounted for −22% (−158,000 to 26,000), −11% (−92,000 to 55,000), and 49% (246,000 to 382,000), respectively (Supplementary Table S4). The pattern found with the claims data was therefore reproduced by the survey data alone.

## 4. Discussion

By linking survey-measured disease burden to claims-based counts of treated patients, this study shows that the rapid growth in younger Korean adults under treatment for hypertension and diabetes is more consistent with increased detection and treatment uptake than with an increase in measured disease prevalence. Between 2010–2012 and 2022–2024, the prevalence of hypertension and diabetes among adults aged 30–49 years did not change, and the population at these ages shrank by 12%, yet the number of treated patients grew as the proportion of people with the condition who were diagnosed and treated rose by about 20 percentage points. For hypercholesterolaemia, the picture is mixed. Measured prevalence doubled, consistent with rising adiposity and with the sharp increase in lipid-lowering medication, which itself enters the prevalence definition, and treatment uptake contributed a similar amount. At ages 50 years and older, growth in the number of patients treated for hypertension and diabetes was driven mainly by growth in the population at those ages, that is, by population ageing, with treatment uptake as a secondary factor.

These results are relevant to the interpretation of growth in chronic disease expenditure. Because treated patients accumulate expenditure for as long as they remain in treatment, the expenditure consequences of the trends documented here may differ from those of a rising disease burden. If the additional treated patients are mainly people whose condition would otherwise have been diagnosed later, the main consequence would be a longer duration of treatment rather than a larger number of people ever treated; if they include people who would otherwise never have been diagnosed, the number of people ever treated would also rise. The present aggregate data cannot distinguish these possibilities, which remain hypotheses requiring cohort data on age at diagnosis and at treatment initiation. Economic analyses show that the per-person cost of diabetes is substantial and sustained over the course of the disease [5,6], and in Korea, participation in cardiovascular health screening has been shown to affect subsequent clinical outcomes and healthcare costs [25]. Although the present study does not estimate expenditure directly, the earlier entry of a growing share of adults in their thirties and forties into long-term treatment has potential implications for lifetime treatment exposure and future expenditure that projections relying on demographic ageing alone [7] do not capture.

Several factors may have contributed to the increase in detection. Korea’s biennial national health screening programme, which includes blood pressure, fasting glucose, and lipid measurement, has extended its coverage to younger adults, including non-employed dependants aged 20–39 years from 2019, and participation has increased [2,26]. Studies of the programme have shown that screening results above diagnostic thresholds lead to confirmatory examination and increased treatment uptake, and that screening participation raises the probability of a first treatment for diabetes [13,27,28]. Clinical guidelines have also moved towards earlier detection: the Korean Diabetes Association recommends diabetes screening from age 35 years in adults [10], and although Korean hypertension guidelines retain 140/90 mmHg as the treatment threshold [11], the 2017 US guideline that redefined stage 1 hypertension at 130/80 mmHg [12] has heightened attention to blood pressure in younger adults. In our sensitivity analysis, increased diagnosis accounted for most, but not all, of the increase in treatment among younger adults, and the proportion of diagnosed people who were treated also rose; this is compatible with wider screening and diagnosis as the larger contributor, together with a greater propensity to treat people once diagnosed, although the ecological design cannot establish either mechanism causally. The pattern is consistent with global evidence that treatment of hypertension has expanded most in high-income countries with universal coverage and organised screening [19], and with the persistently lower awareness and treatment among younger adults reported in the United States, where such programmes are absent [21,22].

The decline in the claims capture ratio also deserves comment. In most groups, the survey-based treated prevalence grew faster than the number of patients recorded under the corresponding principal diagnosis. For hypercholesterolaemia, a declining ratio indicates that claims-based treated prevalence(principal diagnosis) increased less than survey-based treated prevalence; this discrepancy may reflect multimorbidity, principal-diagnosis coding practices, or differences in measurement between the two data sources, for example when lipid-lowering treatment is delivered in a visit recorded under hypertension or diabetes [14]. If coding practice is the main source, the negative capture contributions would imply that claims-based treated prevalence understates the true expansion of treatment, particularly at older ages where multimorbidity is common, but the present data cannot confirm this. Ratios above one among younger adults with hypertension should not be over-interpreted: after allowance for duplicate counting across age groups, the ratio in this group was close to one in the later waves, and the remaining differences in level may reflect differences in treatment definitions between the two sources. More generally, the two sources describe related but distinct populations: the most recent national hypertension fact sheet reports 11.9 million people using hypertension-related care in 2023, compared with an estimated 12.6 million adults with hypertension [29].

This study cannot determine whether earlier and wider treatment of younger adults is beneficial, but the framing of the question matters. Treatment of hypertension and diabetes in early adulthood may reduce cumulative exposure to cardiovascular risk, and young-onset type 2 diabetes in particular carries a more aggressive course than later-onset disease [8,9]. In Korea, the gap in diabetes prevalence between people with and without obesity is largest at ages 30–59 years, and obesity-related diabetes has been described as a distinct high-risk phenotype [30]. The absolute benefit of treatment, however, depends on baseline risk, which is generally low in this age group, whereas lifetime treatment costs are highest for those treated earliest. Earlier diagnosis through screening and overdiagnosis are distinct: the former identifies disease that would otherwise have caused harm before being detected, whereas the latter labels and treats people whose condition would not have harmed them [15]. Lower thresholds and wider screening increase the risk of overdiagnosis and low-value treatment, but aggregate data such as ours cannot separate the two or identify low-value care, which requires individual-level information on baseline risk, treatment intensity, and outcomes; our findings should therefore not be read as evidence of overtreatment. Our results are consistent with a growing share of the additional patients entering treatment in Korea being people in their thirties and forties with newly detected disease, whose severity could not be assessed with these data. The policy question that follows is how to evaluate the value of treating this group, rather than how to restrain aggregate expenditure or attribute it to ageing.

The main strengths of this study are the use of complete national principal-diagnosis patient counts rather than sampled claims, nationally representative examination-based measures of prevalence and treatment, an exact decomposition without residual, explicit propagation of survey sampling error, sensitivity analyses that separate detection from treatment propensity, and robustness checks that include a decomposition using survey data alone. The approach requires only routinely published aggregate statistics and can be applied wherever a health examination survey reports age-specific prevalence and treatment rates and an administrative system reports age-specific treated patients, including countries with national examination surveys such as the United States, the United Kingdom, Japan, and China. Applied over successive survey waves, it offers a simple surveillance indicator for distinguishing growth in disease burden from growth in health-system response.

This study has several limitations. First, the linkage is ecological: survey estimates and claims counts are combined within sex–age cells, and the decomposition is an accounting identity that identifies proximate, not causal, components; statements that treatment uptake rather than prevalence accounts for the observed growth therefore describe consistency with the data rather than a demonstrated causal mechanism. Second, the survey and claims measures of treatment differ in definition; the capture ratio absorbs these differences, and its interpretation as coding behaviour is partly speculative. In particular, the claims category E78 covers a broader range of lipid disorders than the survey definition of hypercholesterolaemia, and the age-specific claims tables count some patients in more than one age group; both affect mainly the level of the capture ratio, and the survey-only decomposition reproduced the main findings. Third, survey estimates for adults in their thirties rest on small numbers of people with each condition, particularly for diabetes; we therefore report combined estimates for ages 30–49 with Monte Carlo intervals, and the intervals for the prevalence component in this age group are wide. Fourth, measurement changes over the period, including the addition of glycated haemoglobin to the diabetes definition in 2011, the change in blood pressure devices in 2020–2021, and a change in laboratory in 2022, may have affected trends, although the published trend tables apply consistent definitions where possible. Fifth, medication use enters the prevalence definitions of all three conditions; for hypercholesterolaemia in particular, part of the measured prevalence increase is itself a consequence of wider lipid-lowering treatment, so the prevalence component for this condition is likely to overstate the change in underlying disease burden and the treatment component to understate the contribution of treatment uptake. Sixth, adults aged 19–29 years were excluded for lack of statistical precision, and the findings should not be extrapolated to this age group. Seventh, the claims statistics are tabulated by review year and averaged over calendar years, whereas the survey waves are defined by examination year, so the two sources are aligned only approximately in time. Eighth, because the study compares sex–age cells rather than individuals, changes in the composition of cells over time, for example in body mass index or healthcare access, could contribute to the observed trends without being captured by any of the four components.

## 5. Conclusions

The number of Korean adults aged 30–49 years treated for hypertension and diabetes grew substantially between 2010 and 2024 despite a shrinking population and unchanged measured disease prevalence at these ages, while the proportion of people with the condition who were diagnosed and treated rose by about 20 percentage points. The expansion of treated hypertension and diabetes population among younger adults therefore is more consistent with increased detection and treatment uptake than with changes in measured disease prevalence. Monitoring of chronic disease burden and expenditure should distinguish these components, and the value of treatment initiated at younger ages should be evaluated directly. Claims-based treated prevalence reported by surveillance systems should be interpreted alongside survey-based prevalence and treatment rates, so that growth in the number of people under treatment is not mistaken for growth in disease burden. In Korea, where both survey and claims statistics are published routinely by age and sex and national fact sheets already present the two side by side [29], an age-specific comparison of survey-based and claims-based treated prevalence in national chronic disease surveillance would make this distinction available on a regular basis.

## Figures and Tables

**Table 1:** Population, survey-measured prevalence, awareness and treatment rates, and claims-based treated patients at the first and last survey waves, by disease and age band.

Disease and Age Band	Wave	Population (Thousands)	Prevalence (%)	Awareness (%)	Treatment (%)	Treated Patients, Claims (Thousands)	Treated Prevalence, Claims (%)	Capture Ratio
Hypertension, 30–49	2010–12	16,819	13.1	36.3	28.9	916	5.45	1.44
	2022–24	14,868	13.5	52.6	48.6	1089	7.32	1.12
Hypertension, 50–69	2010–12	11,326	44.3	73.3	68.1	3097	27.34	0.91
	2022–24	16,123	41.4	80.0	76.9	4380	27.17	0.85
Hypertension, ≥70	2010–12	3683	64.3	85.2	82.6	1579	42.89	0.81
	2022–24	6169	67.3	88.4	86.6	2560	41.50	0.71
Diabetes, 30–49	2013–15	16,627	5.4	44.1	34.7	367	2.21	1.18
	2022–24	14,868	5.5	64.1	56.6	511	3.44	1.10
Diabetes, 50–69	2013–15	12,597	18.8	63.7	58.6	1270	10.08	0.92
	2022–24	16,123	20.0	78.8	75.7	2081	12.91	0.85
Diabetes, ≥70	2013–15	4272	29.3	73.7	68.6	662	15.49	0.77
	2022–24	6169	29.3	84.7	82.0	1239	20.09	0.84
Hypercholesterolaemia, 30–49	2010–12	16,819	8.3	25.0	14.9	295	1.75	1.43
	2022–24	14,868	17.1	41.8	33.9	657	4.42	0.77
Hypercholesterolaemia, 50–69	2010–12	11,326	22.9	58.1	47.9	720	6.36	0.58
	2022–24	16,123	43.1	78.1	71.8	1982	12.29	0.40
Hypercholesterolaemia, ≥70	2010–12	3683	19.4	61.1	52.1	155	4.22	0.42
	2022–24	6169	44.9	91.5	88.8	517	8.38	0.21

Prevalence is the sample-size-weighted mean of annual KNHANES estimates in the wave; awareness and treatment are wave-specific KNHANES estimates among people with the condition, pooled across sexes with weights proportional to the number of respondents with the condition. Claims-based patients and population are three-year averages. Capture ratio = claims-based treated prevalence/(prevalence × treatment rate). Claims-based counts are sums of published 5-year age-group counts and include patients counted in two age groups within a year (Section 2.1).

**Table 2:** Logarithmic mean Divisia index decomposition of the change in claims-based treated patients between the first and last survey waves, by disease and age band (thousands of patients; percentage of total change in parentheses; Monte Carlo 95% intervals in brackets).

Disease and Age Band	Total Change	Population	Prevalence	Treatment Rate	Capture Ratio
Hypertension, 30–49	172	−89 (−52)	−76 (−44) [−180, 30]	530 (308) [378, 686]	−195 (−113) [−375, −4]
Hypertension, 50–69	1284	1435 (112)	−244 (−19) [−423, −66]	492 (38) [336, 655]	−397 (−31) [−638, −163]
Hypertension, ≥70	981	1034 (106)	83 (8) [−1, 168]	104 (11) [45, 167]	−240 (−25) [−346, −136]
Diabetes, 30–49	144	−44 (−30)	−12 (−8) [−101, 70]	241 (167) [140, 344]	−42 (−29) [−175, 91]
Diabetes, 50–69	811	500 (62)	107 (13) [−39, 253]	471 (58) [353, 591]	−265 (−33) [−448, −82]
Diabetes, ≥70	578	347 (60)	−18 (−3) [−112, 74]	174 (30) [115, 239]	73 (13) [−37, 186]
Hypercholesterolaemia, 30–49	362	−49 (−13)	279 (77) [227, 333]	334 (92) [226, 481]	−205 (−57) [−353, −84]
Hypercholesterolaemia, 50–69	1261	451 (36)	744 (59) [673, 818]	492 (39) [410, 577]	−428 (−34) [−540, −318]
Hypercholesterolaemia, ≥70	362	150 (42)	243 (67) [214, 272]	134 (37) [111, 159]	−166 (−46) [−203, −129]

Hypertension and hypercholesterolaemia: 2010–2012 to 2022–2024; diabetes: 2013–2015 to 2022–2024. Contributions are chain-linked over intermediate waves and summed over sexes and 10-year age groups. Population and claims counts are treated as fixed; intervals reflect sampling error in survey prevalence and treatment rates. Contributions can exceed 100% of the total change because positive components are offset by negative components.

## Data Availability

All data used in this study are publicly available: KNHANES trend tables from the Korea Disease Control and Prevention Agency (https://knhanes.kdca.go.kr (accessed on 28 October 2026)), claims-based statistics from the HIRA Healthcare Bigdata Hub (https://opendata.hira.or.kr (accessed on 28 October 2026)), and population estimates from the Korean Statistical Information Service (https://kosis.kr (accessed on 28 October 2026)). The cell-level analytic dataset (extracted survey estimates with standard errors and sample sizes, claims-based patient counts, and population), a record of the cross-check of extracted survey values against earlier editions of the official statistics, and the complete analysis code are provided as Supplementary File S1.

## Supplementary Materials

The following supporting information can be downloaded at https://www.mdpi.com/article/10.3390/healthcare14213729/s1, Table S1: Sensitivity analysis with awareness rate in place of treatment rate; Table S2: Five-component decomposition separating awareness and treatment among the aware; Table S3: Robustness of the decomposition to pooling weights, simulation scale, correlation between prevalence and treatment draws, and duplicate counting in claims; Table S4: Decomposition of the survey-implied treated population without claims data; Table S5: Comparison of LMDI and Shapley decompositions; File S1: Analytic dataset, verification of extracted values, and analysis code.
