# Supplementary material for: Are Younger Korean Adults Getting Sicker or Getting Treated? Decomposing the Growth in Treated Chronic Disease Prevalence into Disease Burden, Treatment Uptake, and Claims Capture, 2010–2024
Source: Healthcare (Basel). 2026 Nov 3;14(21):3729. doi: 10.3390/healthcare14213729 (PMC13762776; doi:10.3390/healthcare14213729)
Supplement: Supplementary file 1 [file healthcare-14-03729-s001.zip › healthcare-4631263-supplementary-Table S1-S5.pdf]

Supplementary Materials

Are Younger Korean Adults Getting Sicker or Getting Treated? Decomposing the Growth in Treated Chronic Disease Prevalence into Disease Burden, Treatment Uptake, and Claims Capture, 2010–2024

Table S1. Sensitivity analysis: decomposition with awareness rate in place of treatment rate (percentage of total change in treated patients).

Disease and age band	Population	Prevalence	Awareness	Capture ratio
Hypertension, 30–49	−51.8	−43.1	214.6	−19.7
Hypertension, 50–69	111.8	−19.1	27.4	−20.0
Hypertension, ≥70	105.5	8.5	7.8	−21.7
Diabetes, 30–49	−30.2	−8.4	131.6	7.0
Diabetes, 50–69	61.6	13.3	47.8	−22.7
Diabetes, ≥70	60.1	−3.3	23.5	19.6
Hypercholesterolaemia, 30–49	−13.4	76.7	54.5	−17.7
Hypercholesterolaemia, 50–69	35.7	58.8	25.7	−20.3
Hypercholesterolaemia, ≥70	41.5	66.9	27.5	−35.9

Here the capture ratio equals claims-based treated prevalence / (prevalence × awareness rate) and therefore absorbs both the step from diagnosis to treatment and the residual claims-to-survey difference (see Table S2).

Table S2. Five-component decomposition separating awareness and treatment among the aware (percentage of total change in treated patients).

Disease and age band	Total change (thousands)	Population	Prevalence	Awareness	Treatment/awareness	Capture ratio
Hypertension, 30–49	172	−51.8	−43.1	214.6	89.6	−109.4
Hypertension, 50–69	1,284	111.8	−19.1	27.4	10.8	−30.8
Hypertension, ≥70	981	105.5	8.5	7.8	2.7	−24.5
Diabetes, 30–49	144	−30.2	−8.4	131.6	33.3	−26.3
Diabetes, 50–69	811	61.6	13.3	47.8	10.0	−32.7
Diabetes, ≥70	578	60.1	−3.3	23.5	6.5	13.2
Hypercholesterolaemia, 30–49	362	−13.4	76.7	54.5	35.0	−52.7
Hypercholesterolaemia, 50–69	1,261	35.7	58.8	25.7	13.1	−33.4
Hypercholesterolaemia, ≥70	362	41.5	66.9	27.5	9.6	−45.5

Identity: $n = N \times P \times A \times (T/A) \times C$, where A is the awareness rate and T/A the proportion of aware people who are treated; the sum of the awareness and treatment/awareness contributions equals the treatment-rate contribution in Table 2 (point estimates). Treatment/awareness

ratio at ages 30–49 years by wave: hypertension 2010–12 0.80, 2013–15 0.88, 2016–18 0.85, 2019–21 0.87, 2022–24 0.92; diabetes 2013–15 0.79, 2016–18 0.87, 2019–21 0.89, 2022–24 0.88; hypercholesterolaemia 2010–12 0.59, 2013–15 0.60, 2016–18 0.66, 2019–21 0.79, 2022–24 0.81.

Table S3. Robustness of the decomposition to pooling weights, simulation scale, correlation between prevalence and treatment draws, and duplicate counting in claims (percentage of total change; Monte Carlo 95% intervals in thousands of patients).

Specification / disease and age band	Total change (thousands)	Population	Prevalence	Treatment rate	Capture ratio
Baseline (survey-n pooling, normal draws, rho=0)					
Hypertension, 30–49	172	−51.8	−43.9 [−180, 30]	307.5 [378, 686]	−113.0 [−375, −4]
Hypertension, 50–69	1,284	111.8	−19.0 [−423, −66]	38.3 [336, 655]	−30.9 [−638, −163]
Hypertension, ≥70	981	105.5	8.4 [−1, 168]	10.6 [45, 166]	−24.5 [−346, −136]
Diabetes, 30–49	144	−30.2	−8.0 [−101, 70]	167.1 [140, 344]	−28.8 [−175, 91]
Diabetes, 50–69	811	61.6	13.1 [−39, 253]	58.1 [353, 591]	−32.6 [−448, −82]
Diabetes, ≥70	578	60.1	−3.1 [−112, 74]	30.1 [115, 239]	12.6 [−36, 186]
Hypercholesterolaemia, 30–49	362	−13.4	77.0 [227, 333]	92.3 [226, 481]	−56.5 [−353, −84]
Hypercholesterolaemia, 50–69	1,261	35.7	59.0 [673, 818]	39.0 [410, 576]	−33.9 [−540, −318]
Hypercholesterolaemia, ≥70	362	41.5	67.1 [214, 272]	37.2 [111, 158]	−45.8 [−203, −129]
Population-weighted pooling					
Hypertension, 30–49	172	−51.8	−48.3 [−188, 22]	307.5 [378, 686]	−108.8 [−367, 0]
Hypertension, 50–69	1,284	111.8	−18.7 [−422, −63]	38.3 [336, 655]	−31.2 [−638, −161]
Hypertension, ≥70	981	105.5	8.6 [0, 170]	10.6 [45, 166]	−24.7 [−347, −139]
Diabetes, 30–49	144	−30.2	−10.8 [−104, 65]	167.1 [140, 344]	−25.7 [−170, 93]
Diabetes, 50–69	811	61.6	12.7 [−43, 250]	58.1 [353, 591]	−32.3 [−444, −78]
Diabetes, ≥70	578	60.1	−4.0 [−117, 71]	30.1 [115, 239]	13.5 [−32, 192]
Hypercholesterolaemia, 30–49	362	−13.4	77.5 [229, 334]	92.3 [226, 481]	−56.7 [−355, −86]
Hypercholesterolaemia, 50–69	1,261	35.7	58.6 [668, 814]	39.0 [410, 576]	−33.5 [−535, −311]
Hypercholesterolaemia, ≥70	362	41.5	66.8 [213, 270]	37.2 [111, 158]	−45.5 [−202, −128]
Unweighted (simple-mean) pooling					
Hypertension, 30–49	172	−51.8	−48.1 [−188, 22]	307.5 [378, 686]	−109.0 [−367, −1]
Hypertension, 50–69	1,284	111.8	−19.1 [−427, −69]	38.3 [336, 655]	−30.7 [−633, −156]
Hypertension, ≥70	981	105.5	9.0 [4, 175]	10.6 [45, 166]	−25.1 [−353, −143]

Diabetes, 30–49	144	−30.2	−10.5 [−104, 65]	167.1 [140, 344]	−26.1 [−172, 93]
Diabetes, 50–69	811	61.6	11.4 [−53, 240]	58.1 [353, 591]	−31.0 [−434, −69]
Diabetes, ≥70	578	60.1	−4.6 [−122, 67]	30.1 [115, 239]	14.1 [−28, 196]
Hypercholesterolaemia, 30–49	362	−13.4	77.3 [228, 333]	92.3 [226, 481]	−56.6 [−354, −85]
Hypercholesterolaemia, 50–69	1,261	35.7	58.5 [667, 813]	39.0 [410, 576]	−33.4 [−533, −310]
Hypercholesterolaemia, ≥70	362	41.5	67.1 [214, 271]	37.2 [111, 158]	−45.9 [−203, −130]
Logit-scale draws					
Hypertension, 30–49	172	−51.8	−43.1 [−177, 29]	305.1 [372, 673]	−111.6 [−369, −2]
Hypertension, 50–69	1,284	111.8	−19.0 [−423, −66]	38.3 [336, 655]	−31.0 [−641, −162]
Hypertension, ≥70	981	105.5	8.5 [−2, 168]	10.7 [45, 168]	−24.6 [−347, −136]
Diabetes, 30–49	144	−30.2	−5.9 [−94, 70]	163.9 [138, 330]	−26.9 [−163, 89]
Diabetes, 50–69	811	61.6	13.0 [−38, 250]	57.9 [352, 588]	−32.2 [−444, −81]
Diabetes, ≥70	578	60.1	−3.3 [−112, 72]	30.1 [116, 240]	12.7 [−35, 186]
Hypercholesterolaemia, 30–49	362	−13.4	76.3 [226, 329]	89.3 [223, 435]	−52.5 [−313, −77]
Hypercholesterolaemia, 50–69	1,261	35.7	58.7 [671, 812]	38.9 [409, 574]	−33.6 [−535, −312]
Hypercholesterolaemia, ≥70	362	41.5	66.7 [213, 269]	37.2 [111, 159]	−45.4 [−201, −128]
Logit draws, corr(P,T)=+0.5					
Hypertension, 30–49	172	−51.8	−43.1 [−177, 29]	304.8 [374, 672]	−110.6 [−404, 34]
Hypertension, 50–69	1,284	111.8	−19.0 [−423, −66]	38.2 [343, 647]	−31.2 [−690, −117]
Hypertension, ≥70	981	105.5	8.5 [−2, 168]	10.7 [46, 167]	−24.7 [−369, −111]
Diabetes, 30–49	144	−30.2	−5.9 [−94, 70]	163.8 [140, 331]	−27.0 [−192, 112]
Diabetes, 50–69	811	61.6	13.0 [−38, 250]	57.9 [352, 586]	−32.3 [−487, −42]
Diabetes, ≥70	578	60.1	−3.3 [−112, 72]	30.1 [114, 239]	12.7 [−59, 214]
Hypercholesterolaemia, 30–49	362	−13.4	76.3 [226, 329]	89.5 [222, 435]	−52.1 [−335, −56]
Hypercholesterolaemia, 50–69	1,261	35.7	58.7 [671, 812]	39.1 [408, 573]	−33.5 [−561, −290]
Hypercholesterolaemia, ≥70	362	41.5	66.7 [213, 269]	37.2 [111, 158]	−45.4 [−209, −120]
Logit draws, corr(P,T)=-0.5					
Hypertension, 30–49	172	−51.8	−43.1 [−177, 29]	304.7 [372, 669]	−109.9 [−323, −52]
Hypertension, 50–69	1,284	111.8	−19.0 [−423, −66]	38.2 [333, 652]	−31.2 [−578, −234]
Hypertension, ≥70	981	105.5	8.5 [−2, 168]	10.8 [47, 164]	−24.6 [−318, −167]

Diabetes, 30–49	144	−30.2	−5.9 [−94, 70]	163.9 [136, 335]	−26.2 [−132, 54]
Diabetes, 50–69	811	61.6	13.0 [−38, 250]	58.0 [351, 594]	−32.4 [−398, −136]
Diabetes, ≥70	578	60.1	−3.3 [−112, 72]	29.9 [111, 239]	12.8 [−4, 156]
Hypercholesterolaemia, 30–49	362	−13.4	76.3 [226, 329]	89.3 [220, 433]	−52.1 [−287, −97]
Hypercholesterolaemia, 50–69	1,261	35.7	58.7 [671, 812]	38.8 [408, 578]	−33.5 [−501, −340]
Hypercholesterolaemia, ≥70	362	41.5	66.7 [213, 269]	37.2 [112, 158]	−45.3 [−192, −137]
Claims counts rescaled for duplicate counting across age groups					
Hypertension, 30–49	155	−51.1	−43.4 [−160, 27]	303.6 [336, 608]	−110.2 [−331, −2]
Hypertension, 50–69	1,149	110.7	−18.8 [−376, −58]	38.0 [298, 581]	−29.7 [−555, −134]
Hypertension, ≥70	878	104.6	8.3 [−1, 149]	10.5 [40, 148]	−23.4 [−300, −113]
Diabetes, 30–49	128	−30.6	−8.1 [−90, 62]	168.6 [125, 308]	−29.8 [−158, 81]
Diabetes, 50–69	723	62.0	13.2 [−35, 227]	58.5 [317, 530]	−33.5 [−406, −78]
Diabetes, ≥70	516	60.3	−3.1 [−101, 66]	30.2 [103, 214]	12.2 [−34, 166]
Hypercholesterolaemia, 30–49	334	−13.8	79.2 [216, 315]	94.5 [214, 456]	−60.6 [−344, −89]
Hypercholesterolaemia, 50–69	1,165	36.5	60.3 [636, 772]	39.8 [386, 544]	−36.8 [−534, −324]
Hypercholesterolaemia, ≥70	335	42.3	68.4 [202, 256]	37.9 [105, 150]	−48.6 [−198, −128]

Baseline: annual prevalence pooled with survey sample-size weights, normal draws bounded below at 0.0001 (prevalence) and 0.001 (treatment), independent draws (B = 2000). Population-weighted pooling uses Statistics Korea mid-year population of the sex–age cell in each year; unweighted pooling uses the simple mean of the three annual estimates. Logit-scale draws use delta-method standard errors, SE(logit p) = SE(p)/[p(1 − p)]. Correlated draws impose corr(P, T) = ±0.5 within each cell and wave. Duplicate-count rescaling multiplies each age-specific claims count by the ratio of the de-duplicated sex-specific total to the sum of the age-specific counts for that disease, year, and sex (factors 0.88–0.89 for I10, 0.89–0.91 for E11, 0.93–0.96 for E78). Medians of percentage contributions are reported, so the components need not sum exactly to 100%.

Table S4. Decomposition of the survey-implied treated population (N × P × T) without claims data (thousands of people; percentage of total change in parentheses; Monte Carlo 95% intervals in brackets).

Disease and age band	Total change	Population	Prevalence	Treatment rate
Hypertension, 30–49	303	−76 (−25.0)	−65 (−21.5) [−158, 26]	445 (147.0) [312, 570]
Hypertension, 50–69	1,858	1,581 (85.1)	−245 (−13.2) [−433, −53]	523 (28.1) [359, 690]
Hypertension, ≥70	1,622	1,375 (84.7)	106 (6.5) [−6, 220]	140 (8.6) [62, 222]
Diabetes, 30–49	155	−38 (−24.9)	−16 (−10.6) [−92, 55]	211 (136.6) [121, 294]
Diabetes, 50–69	1,119	538 (48.1)	86 (7.7) [−60, 239]	496 (44.3) [374, 620]
Diabetes, ≥70	621	431 (69.4)	−33 (−5.4) [−150, 79]	221 (35.6) [148, 302]
Hypercholesterolaemia, 30–49	634	−56 (−8.9)	312 (49.3) [246, 382]	379 (59.8) [275, 484]

Hypercholesterolaemia, 50–69	3,570	1,034 (29.0)	1,569 (44.0) [1,412, 1,736]	967 (27.1) [803, 1,125]
Hypercholesterolaemia, ≥70	2,098	613 (29.2)	971 (46.3) [855, 1,090]	515 (24.6) [435, 590]

The survey-implied number of treated people is the product of population, survey prevalence, and treatment rate in each sex–age cell, summed over cells; logit-scale draws, independent across indicators. Population is treated as fixed, so the interval for the population component reflects only the change in logarithmic-mean weights. Because no claims data enter, this decomposition is unaffected by the claims definitions (principal diagnosis, scope of E78, duplicate counting across age groups) and by the capture ratio.

Table S5. Comparison of LMDI-I and Shapley decompositions (point estimates; percentage of total change in treated patients).

Disease and age band	Population LMDI / Shapley	Prevalence LMDI / Shapley	Treatment rate LMDI / Shapley	Capture ratio LMDI / Shapley
Hypertension, 30–49	−51.8 / −52.4	−43.1 / −43.3	304.2 / 309.3	−109.4 / −113.5
Hypertension, 50–69	111.8 / 111.9	−19.1 / −19.2	38.2 / 38.2	−30.8 / −31.0
Hypertension, ≥70	105.5 / 105.5	8.5 / 8.5	10.5 / 10.5	−24.5 / −24.5
Diabetes, 30–49	−30.2 / −30.7	−8.4 / −7.7	164.9 / 166.2	−26.3 / −27.8
Diabetes, 50–69	61.6 / 61.8	13.3 / 13.2	57.8 / 58.1	−32.7 / −33.1
Diabetes, ≥70	60.1 / 60.1	−3.3 / −3.3	30.0 / 30.0	13.2 / 13.2
Hypercholesterolaemia, 30–49	−13.4 / −13.7	76.7 / 78.0	89.5 / 91.6	−52.7 / −55.9
Hypercholesterolaemia, 50–69	35.7 / 35.9	58.8 / 59.2	38.8 / 39.1	−33.4 / −34.2
Hypercholesterolaemia, ≥70	41.5 / 41.8	66.9 / 67.4	37.1 / 37.7	−45.5 / −47.0

The Shapley decomposition averages the marginal effect of changing each factor over all 4! orderings of the factors and, like LMDI-I, is exact. Both are applied within each sex–age cell and pair of consecutive waves, and summed.
